# Paediatric CT scan usage and referrals of children to computed tomography in Germany-a cross-sectional survey of medical practice and awareness of radiation related health risks among physicians

**DOI:** 10.1186/1472-6963-12-47

**Published:** 2012-02-25

**Authors:** Hiltrud Merzenich, Lucian Krille, Gael Hammer, Melanie Kaiser, Shunichi Yamashita, Hajo Zeeb

**Affiliations:** 1 Institute of Medical Biostatistics, Epidemiology and Informatics, University Medical Center, Johannes Gutenberg-University Mainz, Obere Zahlbacher Str. 69, 55131 Mainz, Germany; 2 Atomic Bomb Disease Institute, Nagasaki University Graduate School of Biomedical Sciences, 1-12-4 Sakamoto, Nagasaki 8528523, Japan; 3 Department of Prevention and Evaluation, Bremen Institute for Prevention Research and Social Medicine (BIPS), Achterstraße 30, D-28359 Bremen, Germany

## Abstract

**Background:**

Computed tomography (CT) is a major source of ionizing radiation exposure in medical diagnostic. Compared to adults, children are supposed to be more susceptible to health risks related to radiation. The purpose of a cross-sectional survey among office-based physicians in Germany was the assessment of medical practice in paediatric CT referrals and to investigate physicians' knowledge of radiation doses and potential health risks of radiation exposure from CT in children.

**Methods:**

A standardized questionnaire was distributed to all paediatricians and surgeons in two defined study areas. Furthermore, the study population included a random sample of general practitioners in the two areas. The questionnaire covered the frequency of referrals for paediatric CT examinations, the medical diagnoses leading to paediatric CT referrals, physicians' knowledge of radiation doses and potential health risks of radiation exposure from CT in children.

**Results:**

A total of 295 (36.4%) physicians responded. 59% of the doctors had not referred a child to CT in the past year, and approximately 30% referred only 1-5 children annually. The most frequent indications for a CT examination in children were trauma or a suspected cancer. 42% of the referrals were related to minor diagnoses or unspecific symptoms. The participants underestimated the radiation exposure due to CT and they overestimated the radiation exposure due to conventional X-ray examinations.

**Conclusions:**

In Germany, the frequency of referrals of children to computed tomography is moderate. The knowledge on the risks from radiation exposure among office-based physicians in our sample varied, but there was a tendency to underestimate potential CT risks. Advanced radiological training might lead to considerable amendments in terms of knowledge and practice of CT referral.

## Background

With the advancement of medical science and technologies in health care, diagnostic imaging techniques and interventional radiological procedures are increasingly used to accurately diagnose a wide range of diseases and injuries. During the past 20 years a rapid increase of computed tomography (CT) use could be observed in many countries. The estimated annual number of CT examinations in the United States rose approximately sevenfold from 2.8 million in 1981 to 20 million in 1995 [1]. In 2005, a total of 8.2 millions CT examinations have been conducted in Germany. The proportion of paediatric CT scans was about 1% [2]. The doses of radiation from computed tomography (CT) are relatively high compared to most conventional X-ray examinations. Thus, CT continues to form a major contribution to the collective diagnostic dose of the population in developed countries [3].

The risk of cancer induction through CT scans performed on children has received special attention. Children are supposed to be at higher risk for developing cancer caused by ionizing radiation compared to adults due mainly to the increased radiosensitivity and a longer lifespan after exposure. Brenner and co-workers [1] estimated the lifetime cancer mortality risk attributable to the radiation exposure from abdomen or head CT in a one year old child based on US CT-practice. In the United States some 600.000 CT scans are performed on children annually. Of these children, approximately 140.000 will eventually die of cancer as adults; 500 cancer cases are estimated to be attributable to radiation exposure from CT in early childhood, corresponding to a risk increase of 0.35%.

The international literature on physicians' knowledge regarding radiation dosages and risks due to computed tomography showed a widespread underestimation of diagnostic radiation doses [4-17]. In Germany, two consecutive studies assessed the knowledge on radiation doses among 124 non-radiologist physicians in a university hospital and in the same geographical region among 137 paediatricians outside and inside hospitals [18,19]. The authors concluded that especially the dose assessment of CT examinations pose substantial difficulties for non-radiologists. Hospital-based paediatricians revealed significantly better results than their office-based counterparts.

In order to investigate the situation in a wider geographical area with urban and rural regions we conducted a cross-sectional survey in two defined study areas in East- and West-Germany with a sizeable sample of office-based physicians. The main aims of the presented survey were:

- The assessment of the current practice regarding frequency and justification of referrals for paediatric CT examinations among German physicians who work in private practices,

- The evaluation of the knowledge of radiation doses and potential health risks of radiation exposure from CT in children among prescribers.

## Methods

The survey was cross-sectional by design with an assessment period of four month (November 2009-February 2010). The study was conducted in two study areas: in West-Germany the cities Bingen-Mainz with the rural vicinity (760,000 inhabitants), in East-Germany the cities Chemnitz-Zwickau with the rural surrounding area (1,300,000 inhabitants) were included.

In a preceeding pilot study all radiologists in the study areas were contacted in order to identify the major prescribers of paediatric CTs. The result of a short telephone-based interview revealed that paediatricians contribute about 44% of all CT referrals of children (surgeons 34%, general practitioners 12%, 10% other specialities). Based on this information our study population included all office-based paediatricians and surgeons in the study areas and furthermore a 50%-random sample of general practitioners. Physicians in hospitals were not considered. In total, the entire study population comprised 811 practitioners (in 806 practices). Study participants were enrolled in the survey in written form by mailing a standardized questionnaire. Non-responders received one written reminder and were then contacted repeatedly in order to obtain a telephone interview.

The 23 item questionnaire was designed to assess the current practice regarding referrals for CT examinations in children (newborn to 16 years of age). In detail, we asked for the frequency of referrals for paediatric CT and the affected age groups. Furthermore, criteria for justification of the CT referral were assessed (reasons for ordering CT/medical diagnoses for referrals, consideration of alternative imaging procedures). We asked if parents were routinely informed about possible health risks. Finally, doctors' knowledge on guidelines and recommendations for paediatric radiology and the ongoing scientific discussion on health risks and paediatric radiation were assessed.

**Figure 1:**
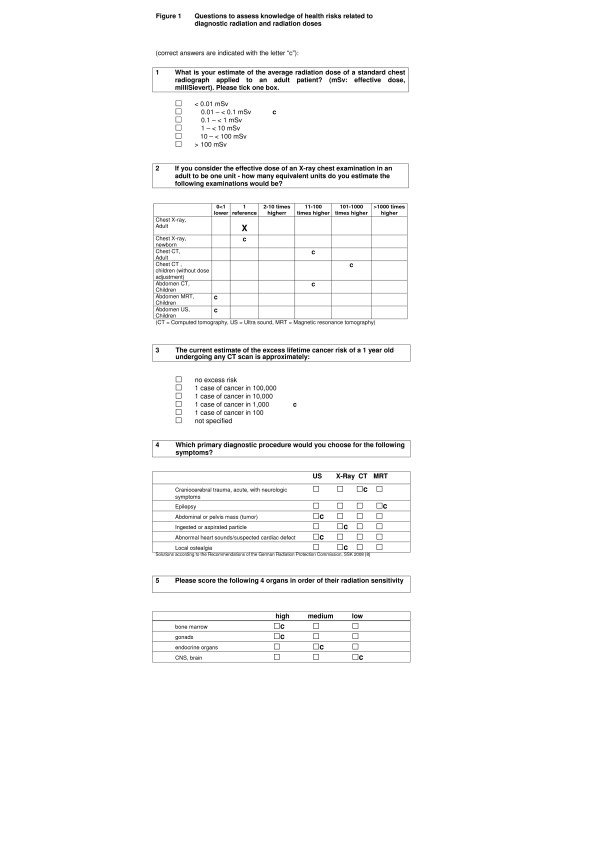
**Questions to assess knowledge of health risks related to diagnostic radiation and radiation doses**.

Five questions aimed at the knowledge regarding potential health risks for children and the effective doses related to CT and alternative imaging methods. Knowledge on radiation doses can be assessed in three approaches. First, participants can be asked for an estimate of the precise effective dose (*What is your estimate of the average effective dose of a standard chest CT in an adult?*). The second possibility is the assessment of dose relations (*Please rank five imaging procedures according to their radiation dose*). Finally, dose equivalents could be addressed (*Please estimate the dose of a procedure equivalent to a standard adult chest radiograph*). In our questionnaire we asked for the precise effective dose of a standard chest radiograph and dose equivalents of CT, MRT procedures in relation to a standard chest radiograph (Figure 1).

SAS for windows, version 9.2 (SAS Institute Inc., Cary, North Carolina) was used for the descriptive analysis. The study was approved by the Ethics Committee of the regional medical chamber of Rhineland-Palatinate (ref. number 837.342.09/6848).

## Results

### The study population

Of the 811 practitioners in the two study regions, 36% (n = 295) completed the mailed questionnaire (n = 106) or took part in a telephone interview (n = 189). The response rate of the contacted paediatricians and surgeons was 52% and 56% respectively. Among the general practitioners the response rate was lower (27%). The distribution of the specialities in the study group is given in Table 1. Among the 295 participants, 28.9% were paediatricians, 25.8% surgeons and 43.6% GPs.

**Table 1:** Distribution of specialities in the study group

Speciality	N	%
Paediatricians	83	28.9

Surgeons	74	25.8

General Practitioners	125	43.6

Other	13	1.7

∑	**295**	**100.0**

### Current practice regarding referrals for paediatric CT examinations

**Figure 2:**
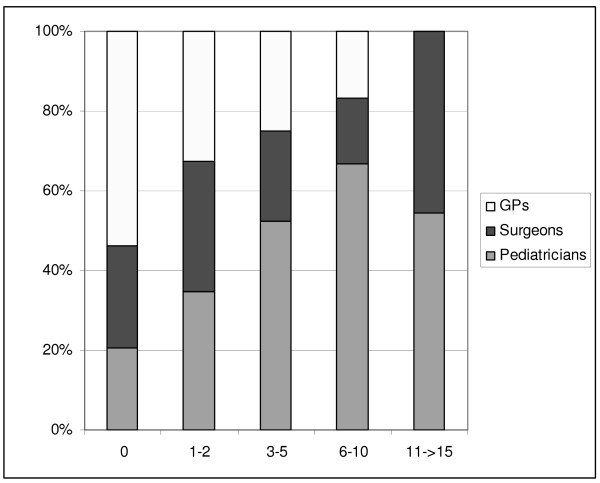
**Frequency classes of CT referrals for children: percentage distribution of doctors' specialities**.

One question aimed at the frequency of CT requests for children over the past year. A total of 59% of the participants indicated that they had not requested any paediatric CT during the last year. A further 30% referred 1-5 children for a CT examination (Table 2). Among those physicians who did not request any CT for children during the last year, approximately 20% were paediatricians (Figure 2). The proportion of paediatricians increased with each frequency class. Only paediatricians and surgeons contributed to the highest category which corresponds to 11 to 15 CT-requests for children in the past year.

**Table 2:** Frequency of annual CT referrals for children

Frequency class	N	%
none	174	59.0

1-2	46	15.6

3-5	41	13.9

6-10	7	2.4

11-15	3	1.0

> 15	8	2.7

n. a.*	16	5.4

∑	**295**	**100.0**

* n.a., no answer

Among those physicians who requested CTs for children (n = 105) nearly 50% of the referrals were related to older children aged 11 to 16 years (Table 3). 41% of the prescribers indicated to routinely consult with a radiologist before sending children for a CT scan. The majority of the physicians who requested CTs considered alternative imaging procedures depending on the given diagnosis or symptom.

**Table 3:** Patient age groups for paediatric CT referrals

Age Group	N	%
0-5	11	10.5

6-10	39	37.1

11-16	52	49.5

n.a.*	3	2.9

∑	**105**	**100.0**

*n.a., no answer

**Figure 3:**
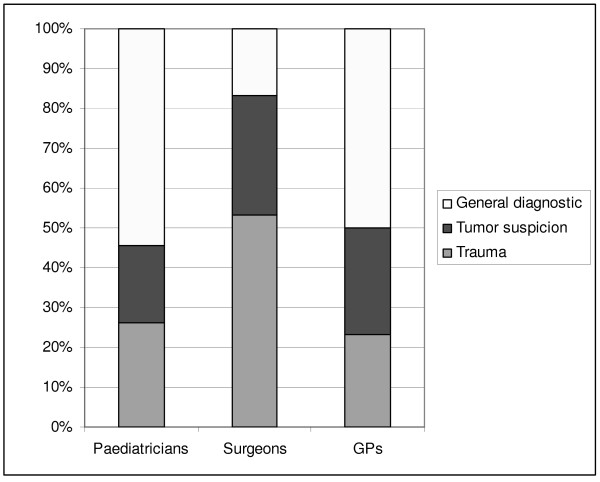
**Percentage distribution of medical indications for requested paediatric CT examinations according to medical specialities**.

We asked the survey participants for the most frequent indications for referrals of paediatric CT. In the group who prescribed CT examinations in the past year (n = 105), the main medical reasons were trauma or a suspected cancer (57%, n = 60). However, 42% of the referrals (n = 44) were for general diagnostic measures. These included diagnoses and symptoms like sinusitis, ambiguous head ache, indigestion/dyspepsia, renal examinations or other unspecific symptoms. Among paediatricians and GPs, general diagnostic measures were a frequent indication for paediatric CT, while "trauma" was the most frequent reason for a CT request among surgeons (Figure 3).

### Knowledge of radiation doses and related health risks for children

Firstly, participants were asked to give an estimate of the average dose of a standard chest X-ray radiograph in an adult. A total of 32.5% answered correctly (0.01-0.1 milliSievert), while 35% overestimated the radiation due to conventional X-ray examinations (Table 4).

**Table 4:** Assessment of the average dose of a standard chest radiograph in an adult

Direction of the answers	N	%
Underestimation	17	5.8

Correct answer	96	32.5

Overestimation	105	35.6

n. a.*	77	26.1

∑	**295**	**100.0**

* n.a., no answer

The second question assessed the dose equivalents of different imaging procedures in comparison to a conventional X-ray radiograph in an adult (If the effective dose of an X-ray chest examination in an adult is being defined as reference, how much higher or lower is the dose from other imaging procedures?) The results are presented in Table 5. The dose of a newborn due to an X-ray image was overestimated by 67.8% of the responders. On the other hand the radiation dose due to a CT scan was underestimated both in adults (47.5%) and children (66.1%). Furthermore 12.9% (n = 38 participants) thought that magnetic resonance tomography involves ionising radiation and 3.1% of the physicians associated ultrasound examinations with ionizing radiation.

**Table 5:** Assessment of equivalent units in relation to an X-ray chest examination in an adult

Imaging procedure	Patients age	Under-estimation	Correct answer	Over-estimation	n.a.*
Chest X-ray	Newborn	16.3%	8.8%	67.8%	7.1%

Chest CT	Adult	47.5%	31.5%	13.5%	7.5%

Chest CT	Child	66.1%	21.7%	4.1%	8.1%

Abdomen CT	Child	25.8%	40.3%	26.8%	7.1%

Abdomen MRI	Child	/	80.0%	12.9%	7.1%

Abdomen Ultrasound	Child	/	90.5%	3.1%	6.4%


* n.a., no answer

Question 3 aimed at the lifetime risk for the development of cancer in a one-year old child after a CT examination. This very specific epidemiological topic was answered correctly by only 3% (approx.1 cancer death per 1,000) and 13% did not respond to this question.

Physicians were asked for the appropriate primary diagnostic procedure for given symptoms (question 4). Compared to the recommendations of the German Radiation Protection Commission [8] a total of 17.8% of the survey participants indicated the correct medical imaging for all symptoms, and a further 26.4% chose the recommended procedure for at least 5 symptoms.

The final question assessed the physicians' knowledge on the radiosensitivity of different organs. The high sensitivity of the bone marrow (gonads) was correctly confirmed by 79% (96%) of the physicians in the study group, whereas the radiosensitivity of brain tissue was overestimated by 2/3 of respondents.

A stratified analysis did not show significant differences between the study regions and only moderate differences between the specialities: surgeons and paediatricians rated slightly better than GPs, for example with regard to the appropriate primary diagnostic procedure for given symptoms (question 4). Finally, no difference could be observed when comparing the two assessment methods: telephone interviews vs. questionnaire.

### Further education and training

40.3% of all physicians in the study group were aware of the recommendations of the German Radiation Protection Commission [8]. A minority (10%) of the study group said that they follow the ongoing scientific debate on the relationship between radiation and possible health risks in children. However, the practitioners indicated an interest to be provided with information concerning radiobiology (19.7%), the relation between cancer and radiation (39.7%), and particular recommendations for paediatric radiology.

## Discussion

The cross-sectional study aimed at the assessment of the current practice regarding CT referrals in children among paediatricians, surgeons and general practitioners in Germany who practice outside of hospitals. Furthermore, knowledge on radiation doses and risks in these groups was investigated.

The 295 participating physicians indicated a moderate frequency of CT referrals for children. This is an expected result since in Germany the proportion of paediatric CT scans in 2005 was about 1% [2] and thus much smaller than in other countries [2,20]. The majority of the referrals for paediatric CT scans appeared to be related to medical problems or diagnostic questions where CT examination are justified [21], but there also seemed to be a fair number of indications where other imaging approaches might be used. A total of 42% of the referrals was ordered for general diagnostic measures like sinusitis, ambiguous head ache, indigestion/dyspepsia, renal examinations or other unspecific symptoms.

The justification for a CT scan request is normally based on the assumption that the benefits are exceeding the risks. CT scanning is advocated to be a sensitive and specific test, and this may lead to an overreliance on CT confirmation for a diagnosis. Moreover, patients commonly expect a detailed examination of their condition including high-tech imaging approaches, which might lower the threshold of physicians to order a CT scan. Worldwide, Japan has the highest number of CT scanners (92.6) per million population in OECD countries, whereas there are 7.5 CT scanners per million population in the United Kingdom, 15.4 in Germany and 32.2 in the USA [22]. A retrospective survey on paediatric CT scan usage was conducted at Nagasaki University Hospital, Japan, in 2004 [23]. The authors selected two common paediatric topics, minor head trauma and acute appendicitis, for a detailed examination of the decision-making process leading to the CT request. A total of 90 children were admitted at the emergency department with minor head trauma. Among them 56 patients (62%) underwent head CT and 76% of cases suspected to be acute appendicitis were referred to CT. The authors emphasized that physicians requesting paediatric CT examinations would do best to involve radiologists more actively. This practice could help clarify the need for a CT examination, consider alternative imaging techniques as ultrasound or MRI, and limit the amount of CT exposure whenever possible. Notably, in our current German survey only of 41% of the prescribers indicated that they consult the radiologist before referring children for a CT scan.

The knowledge on radiation doses in our study group is limited. In detail, we asked for the average effective dose of a standard chest X-ray radiograph in adults. Only 32.5% of the study participants answered correctly. Furthermore, doctors generally tend to overestimate the radiation exposure in children due to conventional X-rays. On the other hand the radiation dose due to a CT scan was underestimated both in adults (47.5%) and children (66.1%). This is in agreement with the published research on knowledge concerning radiation doses among physicians, as mentioned earlier. In most surveys, the physicians were asked to estimate the dose of a procedure of a standard adult chest radiograph. The proportions of correct estimations ranged from 1% in Canada to 22% in the USA. The proportion of physicians underestimating CT doses ranges from 60% up to 87%. More details are given in a recently published systematic review on physicians' knowledge regarding radiation dosages and risks due to computed tomography [24]. Regarding the two earlier German surveys [18,19] our present study supports the interpretation that office-based physicians are not familiar with dose estimations.

Our study has some limitations. The overall response rate was 36% for all medical specialities. The response rate of the general practitioners was only 27%. Hence, the study results are not representative. Furthermore, non-responders from all specialities might have introduced selection bias. Since we do have no information about the medical practice of the non-responders, we cannot exclude the possibility that especially those practitioners who prescribe CT in children more frequently chose not to participate in our survey. Another weakness of the survey is the assessment of medical practice via questionnaire. The validation of the reported data with actual referrals of CT in children was not possible as e.g. claims data per practice could not be accessed centrally. Furthermore, the evaluation of knowledge by asking for quantitative numerical terms may be criticised. Accordingly it might be more appropriate to obtain information on recommended primary examinations, given a specified case scenario. This approach, however, was also part of our investigation (question 4).

Regarding the potential effect of improved training and longer experience on the frequency of CT referrals, the current evidence on the relationship between targeted radiation protection training and knowledge improvements is not strong. For example, a systematic literature review by our group [24] showed that medical specialty as well as duration of professional experience seemed not to be related to knowledge on doses or risks of computed tomography. The data of this survey supports these findings. There was no increasing or decreasing trend in knowledge with the number of years working as a practitioner (data not shown). Nevertheless, apart from tighter quality controls and adherence to referral guidelines, continuous medical education approaches and public campaigns are likely to play a role in the quest for appropriate imaging for paediactric patients and for further dose reduction, both in terms of individual and collective dose. As a current example, the Image Gently campaign, an initiative of the US Alliance for Radiation Safety in Pediatrics, aims to raise awareness of the opportunities to lower radiation doses among radiologists and nuclear medicine physicians who perform imaging exams on children http://www.pedrad.org/associations/5364/ig/.

## Conclusions

While German physicians appear to be relatively restrictive with regard to paediatric CT scanning, our survey shows that there is room for improvement in terms of knowledge and practice of CT referrals, as physicians tend to underestimate radiation risks associated with CT. Overall, adherence to the ALARA (as low as reasonably achievable) principle continues to be important guidance for all physicians in paediatric care.

## Competing interests

The authors declare that they have no competing interests.

## Authors' contributions

All authors contributed to this article and take responsibility for its content. The publication is approved by all authors and tacitly by their institutions' responsible authorities. HM coordinated the survey and wrote the manuscript. LK carried out the data analysis. GH revised the manuscript critically. MK organized the survey and was responsible for the data management. SY revised the manuscript critically and gave input regarding CT use in Japan. HZ was the principal investigator.

## Pre-publication history

The pre-publication history for this paper can be accessed here:

http://www.biomedcentral.com/1472-6963/12/47/prepub
